# Accelerometer-measured physical activity: comparing young adult football players and non-athletic peers

**DOI:** 10.3389/fspor.2026.1797369

**Published:** 2026-03-05

**Authors:** Sasa Jovanovic, Cerasela Domokos, Željko Sekulić, Martin Domokos, Boštjan Šimunič

**Affiliations:** 1 Faculty of Physical Education and Sport, University of Banja Luka, Banja Luka, Bosnia and Herzegovina; 2 Institute for Kinesiology Research, Scientific Research Center Koper, Koper, Slovenia; 3 Faculty of Physical Education and Sport, West University of Timișoara, Timisoara, Romania

**Keywords:** accelerometry, athletes, physical activity, WHO guidelines, transfer effects

## Abstract

While physical inactivity represents a global health concern, the relationship between structured sports participation and overall physical activity patterns remains unclear. This study examined whether football practice enhances adherence to physical activity guidelines and influences lifestyle activity patterns beyond structured practice sessions. Twenty-seven participants [football players (EG) *n* = 16, age 23.4 ± 2.7 years; non-athletes (CG) *n* = 11, age 24.1 ± 3.2 years] wore accelerometers for seven consecutive days. Physical activity parameters including overall physical activity (OPA), physical inactivity (PI), light physical activity (LPA), moderate physical activity (MPA), vigorous physical activity (VPA), and moderate-to-vigorous physical activity (MVPA) were assessed using validated cut-points. Data were analyzed using multivariate GLM procedures, with separate analyses conducted including and excluding structured practice sessions. Both groups exceeded recommendations for MPA (EG: 449.3 ± 98.8 min/week, *p* < 0.001; CG: 414.6 ± 86.4 min/week, *p* = 0.002). However, neither group met VPA recommendations of ≥75 min/week (EG: 60.6 ± 20.5 min/week, 19.3% deficit; CG: 2.8 ± 3.6 min/week, 96.3% deficit). Football players demonstrated 2,064% higher VPA than non-athletes (*p* < 0.001, *η*^2^ = 0.761). Critically, even when practice sessions were excluded, EG maintained significantly higher VPA (13.6 vs. 2.8 min/week, *p* = 0.076) and overall activity levels compared to CG, while exhibiting 46.76% lower LPA (*p* = 0.001, *η*^2^ = 0.376). Structured football participation creates positive transfer effects extending beyond practice contexts, with athletes maintaining higher activity intensities during periods without practice. However, insufficient VPA across both populations highlights the need for targeted interventions to optimize physical activity profiles. These findings support sports participation as a public health strategy while emphasizing the importance of specific high-intensity activity promotion to meet international guidelines.

## 1 Introduction

The health benefits of regular physical activity (PA) are fundamental for overall wellbeing and disease prevention (1). PA represents an essential aspect of a healthy lifestyle and kinesiology, and this study is of great importance for understanding how our body responds to different forms of movement and exercise (2). In contemporary society, physical inactivity has become a widespread problem and is recognized as an independent risk factor with numerous negative health consequences (3). Research has shown that insufficient PA can lead to various chronic health problems, including obesity, diabetes, and heart disease (4). The World Health Organization (WHO) defines PA as “any bodily movement produced by skeletal muscles that requires energy expenditure” (5). The WHO's PA guidelines, updated in 2020, recommend that adults should engage in 150–300 min of moderate-intensity aerobic PA or 75–150 min of vigorous-intensity aerobic PA per week, or an equivalent combination of both (6). WHO recommendations emphasize the importance of both moderate-to-vigorous physical activity (MVPA). Moderate physical activity is defined as activity that “causes slight fatigue and moderately increases heart rate” (7), while vigorous physical activity is activity that “causes significant fatigue and substantially increases heart rate” (8). These definitions are essential when considering the levels of PA among study participants.

The PA of athletes is characterized by significant variations determined by factors such as the type of sport, competitive level, and phase of the practice cycle (9, 10). While endurance athletes practice long-duration high-intensity exercises, strength athletes focus on shorter intensive intervals with longer rest periods (11, 12). Periods of intensive practice alternate with recovery periods through carefully structured periodization (13), with team sports often requiring greater frequency and intensity of activity compared to individual sports (14). During active recovery, athletes incorporate low-intensity activities (15), adjust practice during off-season periods (16) and after injuries (17), while retired athletes often transition to more moderate exercise regimens (18). Collaboration with coaches and sports scientists is key in optimizing PA levels (19, 20) and achieving balance between physical demands and adequate rest for long-term health and well-being of the athlete (21).

Multiple studies have utilized accelerometer-based PA assessment in sports contexts. ActiGraph® is frequently used for measuring PA in adults, providing objective data on activity intensity, steps, and time spent in various activities (22–24). Recent research suggests that the relationship between structured sports practice and maintaining an active lifestyle outside of practice sessions is not straightforward among adult athletes. Although athletes participate in high levels of physical activities during their sports career, research indicates they may not transfer this into regular physical activity after retirement or during periods without practice (25, 26). Interestingly, studies comparing athletes have found that both athletic factors (such as achievements and injuries) and non-athletic factors significantly impact physical activity patterns post-retirement (27).

Previous research has shown mixed results regarding the translation of sports practice to active lifestyle patterns among adult athletes. Studies indicate that athletic identity and engagement in moderate-to-vigorous physical activity during leisure time significantly decreases after retirement from competitive sports (28). While sport participation fosters healthy behavior and better quality of life, remaining physically active later in life is strongly associated with healthy aging, suggesting that practical habits alone do not automatically translate to active lifestyles (29). Research on former professional athletes revealed that maintaining a physically active lifestyle after career termination has a more significant positive effect on health parameters than simply having been an elite athlete (30). This gap between sports participation and lifestyle activity is further supported by Bellettiere et al. (31), who found that higher occupational physical activity may paradoxically lead to increased sedentary behavior during leisure time, suggesting complex compensatory mechanisms that may also apply to athletes. Kettunen et al. (32) demonstrated that despite high overall activity levels, aging athletes show distinct activity patterns with notable periods of inactivity outside structured practice sessions. A comprehensive study by Fogelholm et al. (33) further identified that while athletic practice provides cardiovascular benefits, it does not necessarily establish habitual movement patterns that transfer to activities of daily living. According to Engström's longitudinal research (2008), sport habitus and athletic diversity, rather than competition level, are the critical factors determining long-term physical activity maintenance.

Although the general health benefits of physical activity, particularly from high-intensity training, are well established, the extent to which such structured and guided exercise leads to a more active overall lifestyle remains unclear. Existing research presents inconsistent findings regarding whether athletes sustain higher levels of physical activity outside of organized training sessions. Some studies suggest that sports participation fosters long-term healthy behaviors, while others indicate compensatory increases in sedentary leisure time or reduced activity following athletic careers. Many investigations rely on self-reported physical activity data or fail to distinguish between training-related and non-training-related activity, which limits the reliability of conclusions about general lifestyle patterns. Furthermore, there is a paucity of research directly comparing athletes and non-athletes in terms of adherence to World Health Organization physical activity recommendations using accelerometer-based measurements. Consequently, there is insufficient evidence to determine whether athletes are more physically active than non-athletes when considering total activity time and activity outside of structured training. Addressing this gap with robust data would enhance understanding of the influence of professional sports on the development of lifelong, sustainable physical activity patterns aimed at improving overall health. Previous research comparing physical activities performed outside organized training to those within structured programs has yielded inconclusive results, lacking clear evidence of a strong positive association. Additionally, the current literature contains significant limitations concerning whether the population meets the physical activity levels recommended by the World Health Organization. To address these gaps, this study seeks to obtain objective measures of physical activity in both athletes and non-athletes using accelerometer-based movement assessment.

The primary goal of this study was to objectively compare the levels of physical activity between athletes and non-athletes, with special attention paid to establishing the results obtained with the recommendations of the World Health Organization related to general physical activity. Secondary goals were to determine the difference from moderate to high-intensity physical activity between athletes and non-athletes; to determine the difference in the overall level of physical activity, as well as to determine the differences that would indicate patterns of behaviour when structured training is excluded from the analysis.

## 2 Materials and methods

### 2.1 Participants

A sample size calculation was performed using G Power software. A two-tailed tests with *α* = .05 and statistical power 0.8, an effect size for vigorous physical activity 1.1 (34), yielded a 24 required participants (12 per group). A written informed consent was obtained from *n* = 27 participants (age range: 18–30 years) in accordance with institutional ethics guidelines to objectively monitor physical activity levels. Participants were stratified into two distinct cohorts: an experimental group comprising professional football players from the senior squad of Football Club Krupa (EG; *n* = 16; M = 23.4 ± 2.7 years) and a matched control group of non-athletes (CG; *n* = 11; M = 24.1 ± 3.2 years). The EG consisted of outfield players who met the criteria for highly trained athletes [according to (35)] prior to data collection and were free from injury during the assessment period.

### 2.2 Physical activity assessment

This investigation was conducted as part of a longitudinal monitoring protocol examining health, well-being, and recovery metrics among elite footballers. PA parameters (OPA, PI, LPA, MPA, VPA, MVPA) were measured across a 7-day observation period using accelerometry devices (wGT3X-BT, Actigraph, LLC, Pensacola, FL, USA). Participants wore the accelerometer attached via an elastic strap around the right waist during all waking hours, excluding water-based activities. Subjects maintained a compliance diary documenting device wear time, removal at day's end, and any temporary removals/reattachments throughout the day. Accelerometers sampled movement at 30 Hz, with data subsequently converted to movement counts in 15-second epochs. The protocol maintained ecological validity through non-intervention during the data collection phase, enabling assessment of habitual physical activity patterns during both practice sessions and free-living conditions. Data collection excluded nocturnal sleep periods and the competitive match fixture occurring on Saturday of the observation week.

### 2.3 Data analysis

The accelerometer data were processed using standard methods; all data outside the wearing time were removed, also data of 20 min of consecutive zeros were removed, too. Inclusion criteria for data validation were at least 10 h (>600 min) (23, 36) of wearing time for a valid day and at least 5 days for a valid record, including one weekend day (37, 38). From a valid record, an overall PA (in cpm) was calculated. Furthermore, time spent in PI, LPA, MPA, VPA and MVPA was estimated using cpm-based thresholds: PI <100 cpm; 101 cpm ≤ LPA < 1,048 cpm; 1,048 cpm ≤ MPA < 5,720 cpm; and VPA ≥5,720 cpm (39, 40). The estimated PA levels (MPA and VPA) were compared to the PA guidelines (6), being 300 and 75 min of weekly minutes of MPA and VPA, respectively.

### 2.4 Statistical analysis

Statistical analyses were conducted utilizing SPSS software (version 25.0; IBM Corporation, Armonk, NY, USA). Descriptive statistics comprising means, standard deviations, and 95% confidence intervals were calculated for all measured variables. The normality of data distribution was assessed using the Shapiro–Wilk test and visual inspection of Q-Q plots. Homogeneity of variance was evaluated using Levene's test. The primary analytical framework employed was the General Linear Model (GLM), with statistical significance established at *α* ≤ .05 for all inferential tests.

One-sample t-tests were performed to assess whether physical activity parameters in each cohort deviated significantly from World Health Organization recommendations. Multivariate analysis of variance was used for between-group comparisons (EG vs. CG) in physical activity parameters. Multivariate analysis of variance was also used to examine differences between CG and EG (including practice) and also between CG and EG (excluding practice time). Within the EG, repeated measures analysis of variance was utilized to investigate the differential effects of practice vs. without practicecontexts on physical activity parameters.

Standardized effect sizes were quantified using partial eta squared (*η*^2^) with interpretive thresholds as follows: small (*η*^2^ = .01), medium (*η*^2^ = .06), and large (*η*^2^ = .14) (41).

## 3 Results

Both cohorts exceeded the minimum recommendation for MPA (≥300 min/week). The CG accumulated 514.6 ± 305 min/week [95% CI (352.7, 476.5), *p* = .002], while the EG accumulated 449.3 ± 98.8 min/week [95% CI (396.2, 502.5), *p* < .001]. Conversely, both groups failed to achieve the minimum recommendations for VPA (≥75 min/week). The CG accumulated only 2.8 ± 3.6 min/week [95% CI (0.2, 5.3), *p* < .001], representing a 96.3% deficit compared to recommendations (Table 1). Similarly, the EG, despite accumulating higher VPA than CG (*p* = .013), still failed to meet established recommendations with 60.6 ± 20.5 min/week [95% CI (49.7, 71.4), *p* = .013], falling 19.3% below recommendation. MANOVA revealed differences between EG (with football practice) and CG on PA parameters [Wilks’ *Λ* = 0.185, F (6, 19) = 13.911, *p* < .001, *η*^2^ = .815], indicating a substantial multivariate difference between experimental and control groups across the measured physical activity variables. *post hoc* univariate analyses revealed 2,064% higher VPA and 31.74% lower LPA in EG than in CG (Table 1). Largest differences were found in VPA (*η*^2^ = 0.761).

**Table 1:** Between-group differences in physical activity parameters with practice included in the experimental group.

Variable	CG (M ± SD)	EG (M ± SD)	*p*-value	Partial *η*^2^
OPA (cpm)	296.8 ± 149.8	355.3 ± 63.2	0.178	
PI (min/week)	3,804 ± 648.1	4,197 ± 565.7	0.116	
LPA (min/week)	1,195 ± 346.9	815.6 ± 157.6	0.001	0.379
MPA (min/week)	514.6 ± 305.9	449.3 ± 99.3	0.433	
VPA (min/week)	2.8 ± 3.6	60.6 ± 20.6	<0.001	0.761
MVPA (min/week)	517.3 ± 306.6	509.9 ± 110.1	0.930	

OPA, overall physical activity; PI, physical inactivity; LPA, light physical activity; MPA, moderate physical activity; VPA, vigorous physical activity; MVPA, moderate-to-vigorous physical activity; CG, control group (non-athletes); e.g., experimental group (football players); cpm, counts per minute.

MANOVA revealed differences between EG (after removing football practice) and CG on PA (Wilks’ Λ = .405, *p* = .005, *η*^2^_p_ = 0.595), indicating a moderate-to-large effect of group membership on the composite of physical activity variables. *post hoc* univariate analyses revealed 13.43% higher PI and 46.76% lower LPA in EG than in CG (Table 2). Largest differences were found in LPA (*η*^2^ = 0.376).

**Table 2:** Between-group differences in physical activity parameters with practice removed from the experimental group results.

Variable	CG (M ± SD)	EG without practice (M ± SD)	*p*-value	Partial η^2^
OPA (cpm)	296.8 ± 149.8	248.9 ± 61.4	0.264	
PI (min/week)	3,804 ± 648.1	4,315 ± 571.6	0.046	0.156
LPA (min/week)	1,195 ± 346.9	814.3 ± 163.2	0.001	0.376
MPA (min/week)	514.6 ± 305.9	380.1 ± 92.7	0.110	
VPA (min/week)	2.8 ± 3.6	13.6 ± 18.2	0.076	
MVPA (min/week)	517.3 ± 306.6	393.8 ± 100.5	0.146	

OPA, overall physical activity; PI, physical inactivity; LPA, light physical activity; MPA, moderate physical activity; VPA, vigorous physical activity; MVPA, moderate-to-vigorous physical activity; CG, control group (non-athletes); EG, experimental group (football players).

After removing football practice results from the EG data, we found 42.74% higher PA, 2.2% higher MPA, 0.9% higher VPA and 2.1% higher MVPA in EG with practice than in EG without practice (Table 3).

**Table 3:** Differences in the percentage of time spent at various physical activity intensities between football players with and without practice.

Variable	EG (with practice) (M ± SD)	EG (without practice) (M ± SD)	*p*-value	Partial Eta squared
OPA	355.3 ± 63.2	248.9 ± 61.4	<.001	0.939
PI (%)	75.9 ± 4.2	78.0 ± 4.0	<.001	0.906
LPA (%)	14.9 ± 2.9	14.9 ± 3.0	0.659	
MPA (%)	8.1 ± 1.6	6.9 ± 1.5	<.001	0.816
VPA (%)	1.1 ± 0.3	0.2 ± 0.3	<.001	0.961
MVPA(%)	9.2 ± 1.7	7.1 ± 1.6	<.001	0.901

OPA, overall physical activity; PI, physical inactivity; LPA, light physical activity; MPA, moderate physical activity; VPA, vigorous physical activity; MVPA, moderate-to-vigorous physical activity; EG, experimental group (football players).

## 4 Discussion

This investigation examined physical activity patterns in young adult football players compared to non-athletic peers through the theoretical lens of structured sport participation's impact on health-related behaviors. Our findings revealed significant differences in physical activity distribution supporting contemporary frameworks suggesting that organized sports fundamentally reshape activity intensity profiles rather than simply increasing overall activity volumes (10, 14). The physical activity of athletes is characterized by significant variations determined by factors such as the type of sport, competitive level, and phase of the practice cycle (9), with team sports often requiring greater frequency and intensity of activity compared to individual sports.

Our results provide mixed support for the postulated hypotheses, revealing a more complex relationship between structured sport participation and physical activity patterns than initially anticipated. Both cohorts successfully met WHO recommendations for moderate physical activity (MPA), confirming our first hypothesis. However, both groups demonstrated insufficient vigorous physical activity (VPA), with control group achieving 96.3% deficit and football players group 19.3% deficit below recommendations. This finding is consistent with research by Lee et al. (4), who demonstrated that insufficient physical activity can lead to various chronic health problems, emphasizing the importance of meeting both moderate and vigorous activity recommendations. Football players demonstrated significantly higher physical activity levels than non-athletes, particularly evident in VPA (2,064% higher) and overall activity redistribution during practice periods, confirming our second hypothesis.

Contrary to our third hypothesis, athletes demonstrated more active lifestyles than non-athletes even when practice sessions were removed from analysis. When practice was excluded, CG had higher overall physical activity (248.9 vs. 296.8 cpm) and greater MVPA (393.8 vs. 517.3 min/week), but in the same time EG had more time in VPA (13.6 vs. 2.8 min/week). This suggests that participation in organized sport creates enduring physical activity habits that transfer beyond structured practice sessions, refuting our initial hypothesis and indicating significant “spillover” effects from structured football practice to physical activity outside practice contexts. These findings contrast with previous research by Sorenson et al. (25) and Witkowski & Spangenburg (26), who suggested that athletes may not transfer high activity levels into regular physical activity during non-practice periods. Our results are in line with Cengizoğlu, Keskin & Kabasakal (42), who found that national-level athletes exhibit progressively healthier lifestyle habits as they advance in their sports careers. This trend underscores the importance of implementing policies that support health-promoting behaviors to sustain athletic performance.

These transfer effects may occur because sport helps individuals develop physical skills and fitness, builds confidence and motivation to be active, creates supportive social connections, and increases awareness of the importance of health (28, 43). According to Engström's longitudinal research (2008), sport habitus and athletic diversity, rather than competition level, are critical factors determining long-term physical activity maintenance. The finding that football players maintain 385% higher VPA (13.6 vs. 2.8 min/week) even without practice sessions suggests that structured sport participation creates lasting behavioral adaptations extending beyond the immediate practice environment. This aligns with research by Bäckmand et al. (29), who found that while sport participation fosters healthy behavior and better quality of life, remaining physically active later in life is strongly associated with healthy aging, though they noted that practice habits alone do not automatically translate to active lifestyles. Our findings suggest a more positive transfer effect may occur during active participation periods.

Very interesting are the data obtained in the analysis of physical activity in athletes when the exercise period is excluded. Namely, the experimental group (EG) achieved 46.76% less light physical activity (LFA) than the control group (CG). This pattern of behavior could rather reflect behavioral adaptation caused by exercise, rather than a simplified reduction in total physical activity. Long-term exposure to structured high-intensity exercise may cause such regulation of athletes’ activity, which would lead to an increased volume of moderate and intense physical activities during exercise, which would consequently lead to a lower level of low-intensity physical activity, in periods without exercise. The tendency of increased efficiency of athletes on the one hand-professional side, consolidates physical activity into shorter, but more purposeful and intense intervals, which leads to an increased physiological and/or perceptual need for passive recovery. The obtained results indicating compensatory redistribution of activity coincide with the concept compensation of physical activity described by Beletjer et al. (32), who indicated that increased physical activity at work can paradoxically lead to increased sedentary and behavior during leisure time. If this principle were applied to athletes, it could be said that high training loads can causally lead to increased absence of light intensity activity, without talking about its complete absence. A similar claim was confirmed by Kettunen et al. (32), who indicated that athletes show different activity patterns characterized by occasional inactivity outside of structured exercise, that these begin to form during active competitive years. Observed from the perspective of training theory, the resulting redistribution of activity coincides with the principles of periodization (9), with the idea of consciously alternating intense physical engagement and recovery necessary for physiological adaptation and optimization of overall performance. All the data obtained indicate that a positive transfer effect, whereby the significant impact of a structured exercise process on shaping longer-term behavioral regulation of physical activity intensity, rather than an increased impact on total volume at all intensity levels.

Contrary to theoretical expectations that sport participation would facilitate complete adherence to international physical activity recommendations (6), our results revealed a more nuanced reality. While both cohorts successfully met moderate physical activity thresholds, they demonstrated insufficient vigorous physical activity—a pattern consistent with global young adult activity trends despite the established neurophysiological benefits of high-intensity exercise (1, 3). Contemporary society faces widespread physical inactivity, which has become recognized as an independent risk factor with numerous negative health consequences, as documented by Kohl et al. (3). The substantial between-group difference in VPA confirms our hypothesis regarding intensity distribution differences while revealing a significant limitation: even structured football participation failed to generate sufficient VPA to meet international guidelines. This observation aligns with Van Hoye et al.'s (44) cross-European findings on activity variability among youth footballers and suggests that current practice methodologies may require recalibration to prioritize higher-intensity stimuli. Our findings also support research by Fenton, Duda, & Barrett (45), who documented inter-participant variability in daily physical activity among male youth sport footballers, emphasizing the complexity of activity patterns in this population.

These findings extend the work of Schlechter et al. (46), who reported that less than half of youth sports time involves MVPA, and highlight the need for targeted interventions to increase VPA across both populations. As noted by Bull et al. (6) in the updated WHO guidelines, children and adolescents should incorporate VPA at least three days per week. Our results suggest that even organized sports participation may not guarantee sufficient VPA to meet these international recommendations. The importance of achieving adequate physical activity levels is further emphasized by the World Health Organization's definition of physical activity as “any bodily movement produced by skeletal muscles that requires energy expenditure” (5), with updated 2020 guidelines recommending specific durations and intensities for optimal health benefits. Research by Teramoto & Bungum (30) revealed that maintaining a physically active lifestyle after career termination has a more significant positive effect on health parameters than simply having been an elite athlete, underscoring the importance of establishing sustainable activity patterns during active participation years.

For coaches and sports scientists, these findings suggest incorporating additional high-intensity practice components to ensure VPA recommendations are met, while recognizing that current practice approaches successfully promote transfer of activity habits beyond structured sessions. The positive spillover effects identified support continued emphasis on sport participation as a public health strategy, while highlighting the need for targeted VPA enhancement (20). Collaboration with coaches and sports scientists is key in optimizing physical activity levels (19, 20) and achieving balance between physical demands and adequate rest for long-term health and well-being of the athlete (21). For public health practitioners, structured sport demonstrates clear benefits for physical activity patterns both during and outside practice periods. However, the insufficient VPA across both groups indicates need for complementary approaches combining the advantages of structured sport with specific high-intensity activity promotion. The evidence of positive transfer effects suggests that sport participation creates lasting behavioral changes extending beyond immediate practice contexts, supporting investment in youth sport programs while emphasizing the importance of designing practice that maximizes both immediate and long-term activity benefits. This is particularly relevant given that research by Fleischman et al. (27) found that both athletic factors (such as achievements and injuries) and non-athletic factors significantly impact physical activity patterns, emphasizing the multifaceted nature of activity maintenance.

Several methodological aspects merit consideration when interpreting our findings. The use of validated ActiGraph accelerometry provided objective measurement of physical activity parameters, addressing limitations of self-report methods noted by Dyrstad et al. (22), who documented significant discrepancies between self-reported and objectively measured physical activity. Our approach aligns with recommendations by Duncan et al. (47) regarding the convergent validity of ActiGraph accelerometers for estimating physical activity in various populations. However, as highlighted by Migueles et al. (24), accelerometer data accuracy depends on device settings and wearing position, which may influence intensity classification. Our analytical framework employed predetermined accelerometer count thresholds following the methodology of Watson et al. (48) for activity intensity classification. This approach enhances comparability with existing literature but introduces inherent limitations in capturing the full complexity of movement patterns in team sports. The 7-day observation period captured a representative sample of weekly activity patterns, though the exclusion of the competitive match fixture from analysis means our results likely underestimate total weekly vigorous activity among football players. Additionally, potential seasonal variations in physical activity were not accounted for, which may affect the generalizability of findings across different times of the year. Sample size limitations, particularly in the control group (*n* = 11), may have reduced statistical power for detecting smaller between-group differences. Furthermore, the cross-sectional design precludes causal inferences regarding the effect of football participation on physical activity patterns.

Future research should extend these findings by examining physical activity patterns across multiple sports disciplines, as the activity characteristics of football may not generalize to other individual or team sports, as suggested by Hawley et al. (11) and Stone et al. (12), who documented sport-specific physiological adaptations to different practice regimens. Longitudinal designs would help clarify whether observed differences reflect practice adaptations or selection effects, where naturally more active individuals gravitate toward sports participation. Investigation of psychological factors mediating the relationship between structured practice and physical activity behaviors would provide valuable insights for intervention development. Additionally, exploring the interaction between practice periodization and daily activity patterns as described by Issurin (13) could inform more effective strategies for maintaining healthy activity levels throughout competitive seasons. As emphasized by Fyfe et al. (15), understanding the role of recovery and active recovery in maintaining optimal physical activity patterns is crucial for comprehensive activity programming. Given the insufficient vigorous activity observed in both groups, experimental studies testing targeted interventions to increase high-intensity engagement would be particularly valuable. Such interventions might leverage principles from sports practice as described by Mujika and Padilla (10) to enhance physical activity programs for the general adolescent population. The challenge of transitioning from structured to unstructured activity contexts (18, 49), among athletes, represents another promising avenue for future investigation.

## 5 Conclusion

In conclusion, this study demonstrates that structured football participation has a complex and largely positive impact on young adult physical activity patterns. Although both young adult football players and non-athletic peers exceed WHO recommendations for moderate physical activity, structured football practice significantly enhances engagement in vigorous physical activity while demonstrating positive transfer effects to periods without practice. The most significant finding is that football players maintain substantially higher physical activity levels even outside practice sessions, refuting our initial hypothesis and indicating that organized sport participation creates enduring behavioral adaptations extending beyond immediate practice contexts.

The pronounced redistribution of activity intensities during practice periods, combined with sustained higher activity levels during periods without practice, underscores the multifaceted benefits of organized sports participation for promoting health-enhancing physical activity patterns. However, the insufficient vigorous physical activity across both groups and the compensatory reduction in light physical activity among athletes highlight the need for targeted interventions to optimize physical activity profiles in young adult populations. These findings contribute to our understanding of how organized sports shape daily activity patterns and provide a foundation for developing more effective physical activity promotion strategies tailored to both athletic and non-athletic young adult. The evidence of positive transfer effects supports continued investment in youth sport programs while emphasizing the importance of designing practice approaches that maximize both immediate performance and long-term health benefits. The health benefits of regular physical activity are fundamental for children and young adult s’ wellbeing, and our results offer practical insights for maximizing these benefits through strategic activity programming and sport-based interventions.

## Data Availability

The raw data supporting the conclusions of this article will be made available by the authors, without undue reservation.
